# A new end‐user–oriented and dynamic approach to post‐disaster resilience quantification for individual facilities

**DOI:** 10.1111/risa.17637

**Published:** 2024-08-23

**Authors:** Gemma Cremen

**Affiliations:** ^1^ Department of Civil, Environmental and Geomatic Engineering University College London London UK

**Keywords:** bottom‐up stakeholder‐driven decision making, dynamic functionality importance, post‐disaster recovery, resilience

## Abstract

Community recovery from a disaster is a complex process, in which the importance of different types of infrastructure functionality can change over time. Most of the myriad of metrics available for measuring disaster resilience do not capture the dynamic importance of functionality explicitly, however. This means that very different recovery trajectories of a given infrastructure can correspond to the same resilience value, regardless of variations in its utility over time. While some efforts have been made to integrate features of time dependency into individual facility resilience quantification, the resulting metrics either capture only a limited set of temporal instances throughout the post‐disaster phase or do not offer a way to prioritize time steps in line with variations in the importance of facility functionality. This study proposes a novel, straightforward metric for component‐level post‐disaster resilience quantification that overcomes the aforementioned limitations. The metric involves a dynamic weighting component that enables stakeholders to place varying emphasis on different temporal points throughout the recovery process. The end‐user–centered approach to resilience quantification facilitated by the metric allows for flexible, context‐specific interpretations of infrastructure functionality importance that may vary across different communities. The metric is demonstrated through a hypothetical case study of infrastructure facilities with varying degrees of importance across the post‐disaster recovery period, which showcases its versatility relative to a previously well‐established measurement of component‐level resilience. The proposed metric has significant potential for use in stakeholder‐driven approaches to decision making on critical infrastructure (as well as other types of built environment) recovery and resilience.

## 1 INTRODUCTION

The need for effective disaster resilience is well established in the literature (Tiernan et al., 2019) and promoted widely across leading international agencies, such as the World Bank and the United Nations (Mochizuki et al., 2018). There is no explicit consensus on the definition of the concept of resilience (Cai et al., 2018), which features across a range of different disciplines including ecology and child psychology (Ayyub, 2014). However, in the context of disasters and communities, the term is broadly captured by the following United Nations Office for Disaster Risk Reduction (UNISDR) explanation: *“A resilient city is characterized by its capacity to withstand or absorb the impact of a hazard through resistance or adaptation, which enable it to maintain certain basic functions and structures during a crisis, and bounce back or recover from an event”* (Johnson & Blackburn, 2012).

Implicit in this interpretation of disaster resilience (particularly through the word “certain”) is the idea that the importance of post‐disaster functionality in a given infrastructure (facility) may change over time. Some facilities, like shelters and hospitals or (more fundamentally) those related to electric power systems, are critical to the emergency response phase and should be immediately functional for maintaining basic needs (e.g., Hassan & Mahmoud, 2018; Cimellaro et al., 2010; Vecere et al., 2017; Eyer & Rose, 2019; Wing & Rose, 2020). On the other hand, other services, such as those related to education, are not required to operate so soon after a disaster; in fact, the reopening of schools often marks the transition from response to recovery efforts (Scott et al., 2023). In addition, the importance of functionality in different facilities at a certain point in time can vary across neighborhoods (Dong et al., 2021). For instance, immediate operation of food assistance services may be critical for low‐income communities, but not necessary for high‐income groups that have sufficient pre‐existing resources to cope without these facilities for a certain period of time.

Yet, the vast majority of existing metrics for individual facility (i.e., component‐level) resilience do not capture the dynamic nature of post‐disaster functionality importance (Hosseini et al., 2016). For instance, the resilience triangle measurement proposed by Bruneau et al. (2003), which is perhaps the most well‐known and widely used metric in this context, can produce the same resilience result for very different functionality trajectories because each time instant is treated equally. Thus, a hospital that has minimal functionality in the critical emergency phase but recovers quickly thereafter could have identical resilience to a similar facility that has significantly more capacity to deal with emergency casualties but recovers to a fully operational status more slowly. This limitation of the Bruneau et al. (2003) metric was identified and addressed by Zobel (2011), Zobel and Khansa (2014), and Chang and Shinozuka (2004), but the resulting approaches only focus on functionality at a finite number of temporal instances (i.e., the beginning and end of recovery processes), such that the importance of performance in intervening periods cannot be accounted for.

While the literature does contain time‐dependent component‐level metrics that enable disaster‐related resilience to be examined and/or distinguished for any temporal instance of interest (e.g., Henry & Ramirez‐Marquez, 2012; Rose, 2007), there has been no attempt to explicitly prioritize (weight) time steps in line with the dynamic importance of facility functionality. Time‐dependent weighting functions have been introduced in the system resilience domain, reflecting the relative importance of functionality in one type of facility or component over another (Ghorbani‐Renani et al., 2020; Sharma et al., 2018; Zhang et al., 2021). However, these types of metrics still treat all time steps with equal importance for a system composed of only one facility or component.

This study addresses the crucial gap identified in the state‐of‐the‐art, by proposing a novel component‐level resilience metric that enables varying emphasis to be placed on different temporal points throughout the recovery process. The dynamic nature of infrastructure functionality importance is specifically captured through a time‐dependent weighting component that should be calibrated in consultation with relevant end users (e.g., resilience planning committees, infrastructure managers, local government authorities). This end‐user–oriented feature of the proposed metric has a number of advantages. First, it allows for flexible, context‐specific interpretations of recovery importance for different infrastructure, addressing possible intercommunity disparities in post‐disaster needs. Second, stakeholder participation in the post‐disaster planning process can lead to greater awareness of related challenges and higher confidence of being able to address them (Chandrasekhar, 2012). Ultimately, end‐user involvement results in better informed decision making (e.g., Komendantova et al., 2014), which is the final goal of any resilience assessment.

The rest of the article is organized as follows. Section 2 introduces the proposed resilience metric, and explains how it may be adapted to consider a system of interdependent facilities. The metric is then demonstrated for a set of hypothetical infrastructure facilities and stakeholders in Section 3. The article ends with a discussion and conclusions in Sections 4 and Section 5, which include a commentary on the utility of the metric and its potential application to infrastructure recovery decision making.

## 2 PROPOSED METRIC

The proposed resilience metric provides a weighted average value of normalized functionality Q(t) for an individual facility between two time instances of interest, $t_{0}$ (typically the time at which the disaster occurs) and $T_{RE}$ (corresponding to some subsequent point in the post‐disaster phase, which may or may not align with the restoration of full functionality in the facility and could be disaster‐specific). Q(t) can be interpreted as a dynamic measurement of facility performance, where the minimum value of 0 corresponds to a complete loss in service, the maximum value of 1 indicates a fully operational facility, and an intermediate value represents a proportional degradation in complete functionality (Bruneau et al., 2003). The metric can be expressed as:

(1) $$R=\frac{\int_{t_{0}}^{T_{RE}}Q\left(t\right)w\left(t\right)dt}{\int_{t_{0}}^{T_{RE}}w\left(t\right)dt},$$

where w(t) is a context‐specific weighting that reflects the relative importance of functionality at time t and ranges intuitively in value from 0 to 1. w(t)=1 indicates that functionality is as important as at other times when functionality is expected/required and w(t)=0 is used when there is full preference for functionality at other times (i.e., functionality at t is not necessary).

Note that Q(t) and w(t) can correspond to a given functionality (rather than a facility), in the case of one facility that performs different functions over time. For instance, schools may be used as emergency shelters in the immediate aftermath of a disaster, before returning to their primary educational purpose later on in the recovery phase (e.g., Hassan et al., 2020).

### 2.1 Quantifying w(t)

w(t) is characterized from discussions with relevant facility stakeholders, which may involve reference to recovery goals set in community resilience plans (e.g., Scott et al., 2023; Poland, 2009). It could be determined using any decision‐making methodology that facilitates the articulation and modeling of preferences. I propose that w(t) is quantified based on concepts from the PROMETHEE (Preference Ranking Organisation Method for Enrichment Evaluation Method) multicriteria decision‐making approach (Vincke & Brans, 1985). For each $C_{j}$ criterion under consideration, PROMETHEE involves defining a preference function $P\left(A_{k},A_{p}\right)=p\left(x\right)\in \left\{0,1\right\}$ for alternative $A_{k}$ over $A_{p}$ based on the difference in their associated values $x=a_{k}-a_{p}$. A number of standard p(x) functions have been established for different decision‐making contexts, which are summarized in Table 1. PROMETHEE is suggested because p(x) assumes the same range of values as that proposed for w(t), the p(x) standard functions may be intuitively linked with the concept of w(t) (details to follow), and from a practical perspective, p(x) may be derived using relatively straightforward questioning of stakeholders involving pairwise comparison (e.g., Guitouni & Martel, 1998). However, alternative methods (such as multiattribute utility theory) could be used as long as the corresponding preference formulation can be mapped to w(t) and the mode of eliciting preferences from stakeholders is deemed appropriate for the context of interest.

**TABLE 1:** Types of typical preference functions p(x) used in the Preference Ranking Organisation Method for Enrichment Evaluation Method (PROMETHEE) method.

Name	p(x)	Explanation
Usual preference function	$\left\{\begin{array}{cc} 0, & \text{if}\;|x|=0\\ 1, & \text{otherwise} \end{array}\right.$	$A_{k}$ is fully preferred over $A_{p}$ if there is any difference in $a_{k}$ and $a_{p}$; there is no preference otherwise.
U‐shape preference function	$\left\{\begin{array}{cc} 0, & \text{if}\;|x|\leq q\\ 1, & \text{otherwise} \end{array}\right.$	$A_{k}$ is fully preferred over $A_{p}$ once the difference between $a_{k}$ and $a_{p}$ reaches a threshold value q; there is no preference otherwise.
V‐shape preference function	$\left\{\begin{array}{cc} 0, & \text{if}\;|x|=0\\ \frac{\left|x\right|}{p}, & \text{if}\;|x|\leq p\\ 1, & \text{otherwise} \end{array}\right.$	Preference for $A_{k}$ over $A_{p}$ linearly increases from 0 to 1 in line with |x|, such that full preference for $A_{k}$ is achieved once the difference reaches a threshold value p.
Level preference function	$\left\{\begin{array}{cc} 0, & \text{if}\;|x|\leq q\\ \frac{1}{2}, & \text{if}\;q\leq |x|\leq p\\ 1, & \text{otherwise} \end{array}\right.$	There is no preference between $A_{k}$ and $A_{p}$ if the difference between $a_{k}$ and $a_{p}$ is below a threshold value q. For larger values of |x|, there is weak preference for $A_{k}$ over $A_{p}$ until |x|=p, when full preference for $A_{k}$ is achieved.
Linear preference function	$\left\{\begin{array}{cc} 0, & \text{if}\;|x|\leq q\\ \frac{|x|-q}{p-q}, & \text{if}\;q\leq |x|\leq p\\ 1, & \text{otherwise} \end{array}\right.$	There is no preference between $A_{k}$ and $A_{p}$ if the difference between $a_{k}$ and $a_{p}$ is below a threshold value q. For larger values of |x|, there is linearly increasing preference for $A_{k}$ over $A_{p}$ until |x|=p, when full preference for $A_{k}$ is achieved.
Gaussian preference function	$\left\{\begin{array}{cc} 0, & \text{if}\;|x|=0\\ 1-e^{-\frac{x^{2}}{2s^{2}}}, & \text{otherwise} \end{array}\right.$	Preference for $A_{k}$ over $A_{p}$ monotonically increases with |x|, with the level of increase controlled by the inflexion point defined from s.

In this case, $A_{k}$ and $A_{p}$ are considered analogous to the functionality of the facility at time $t_{0}^{*}$ (i.e., a time when functionality is expected/required, just before or after the disaster) and t, respectively, and the criterion of interest can be thought of as their time of occurrence, such that $a_{k}=t_{0}^{*}=0$, $a_{p}=t$, and x=0−t=−t. Then, $w\left(t\right)=1-P\left(A_{k},A_{p}\right)=1-p\left(x\right)$. This means that w(t)=1−p(x)=1−0=1 when a stakeholder is indifferent to (i.e., places equal importance on) functionality at both times, whereas w(t)=1−p(x)=1−1=0 when they strongly prefer having functionality at time $t_{0}^{*}$ than at t. For many applications where the importance of functionality remains stable (e.g., in the case of a healthcare facility) or increases over time from a low point after the disaster (e.g., in the case of an educational facility), $t_{0}^{*}=t_{0}^{-}$, which refers to the final instant of “normal times” just before the disaster strikes. For other applications where the importance of functionality decreases over time (e.g., in cases where emergency facilities are deployed on a temporary basis and later become unnecessary), $t_{0}^{*}=t_{0}^{+}$, which refers to the moment directly after the disaster strikes. In the case where an emergency facility becomes unnecessary, w(t)=0 for the time when conditions are restored to a more permanent state.

The first step in characterizing w(t) is to determine $t_{0}^{*}$ according to whether the importance of functionality increases, remains stable, or decreases in the post‐disaster period. Then, the parameters q, p, and s that define p(x) (see Table 1) should be assessed, through discussions with stakeholders that involve successive approximations of their values until satisfactory ones are achieved. For instance, in the case of increasing functionality preference over time, the stakeholder should initially be presented with: (1) a very small potential value for q that is progressively increased until $q=t_{q}$, the first time instance at which the stakeholder no longer holds a full preference for functionality at $t_{0}^{*}$; (2) a very large potential value for p that is gradually decreased to $p=t_{p}$, the farthest time instance at which the stakeholder has some preference for functionality at $t_{0}^{*}$; and possibly—if the stakeholder has managed to define p and q but is not entirely happy with their concept—(3) a value of s that is increased from q to a maximum of p, until the Gaussian curve reflects the functionality preferences of the stakeholder.

**FIGURE 1:**
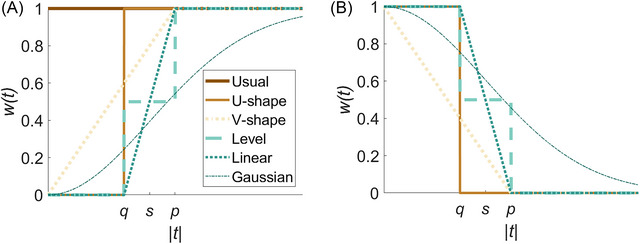
Sample w(t) trajectories corresponding to each p(x) functional form, in the case of (A) increasing (or stable) and (B) decreasing preference for functionality with time since the disaster. Note that p, q, and s are defined in Table 1.

These stakeholder discussions and the type of facility of interest will determine the appropriate functional form of p(x) to be selected from Table 1. If q cannot be defined, the choices for p(x) are limited to the usual or V‐shape preference functions. If p cannot be defined (i.e., in cases where the importance of functionality remains constant with time), then p(x) is the usual preference function. If p and q are not entirely satisfactory for the stakeholder, then p(x) is the Gaussian function (which represents a certain monotonic increase or decrease in w(t) that is defined in line with s). The U‐shape function (which represents a sudden change in functionality preference from w(t)=0 to w(t)=1, or vice versa, at q) may be useful in cases where facilities become redundant in the longer term. The V‐shape function (which represents consistently increasing values of w(t) from 0 to 1 until p, or vice versa) would be useful if small time steps yield notable differences in the importance of functionality. The level preference function (which provides w(t) values of 1 or 0 until t=q and t>p, and w(t)=0.5 from t=q until t=p) would be suitable if there is a consistently weak degree of preference for functionality at certain times. The linear preference function (which represents consistently increasing values of w(t) from 0 to 1 after t=q until t=p, or vice versa) would be appropriate if functionality gradually decreases in importance and becomes redundant after some length of post‐disaster period. Figure 1 provides sample w(t) values associated with each p(x) functional form.

The expression for w(t) associated with a given facility may depend on the severity of a disaster. For instance, the importance of functionality in an emergency shelter may last longer for events that cause substantial residential damage than those that have minimal effect on a region's housing stock. On the other hand, the time at which educational facilities should reach full capacity may be later for high‐impact events that require a protracted post‐disaster emergency phase. w(t) should also account for any resilience tactics (e.g., Rose & Huyck, 2016) associated with the facility of interest that can be used to supplement or as a substitute for its functionality over a prescribed period of time. For example, w(t) may be zero for an industrial premises during the time period that the associated business can operate with employees working from home (e.g., Cremen et al., 2020).

### 2.2 Comparison with existing metrics

#### 2.2.1 Component‐level metrics

R is a modified version of the straightforward well‐known resilience triangle concept (herein referred to as $R^{*}$) proposed by Bruneau et al. (2003) and subsequently updated by Cimellaro et al. (2005). The inclusion of the integral on the denominator of R normalizes the metric, analogous to the $\frac{1}{T_{LC}}$ component of the formulation proposed by Cimellaro et al. (2005), where $T_{LC}$ refers to a specific time period of interest (equivalent to $T_{RE}$ in Equation (1)). R reduces to $R^{*}$ for w(t)=C, where C is some constant between 0 and 1, that is, the two metrics are equivalent when an equal amount of importance is placed on the functionality of the facility of interest across the time $\left\{t_{0},T_{RE}\right\}$. This may arise in the case of some facility that is required to successfully operate under all conditions, such as a critical bridge in a road network.

#### 2.2.2 System‐level metrics

Individual facilities are just one component within a complex set of intricately interdependent physical, social, and economic infrastructure that typically comprise a community (e.g., Koliou et al., 2020; Wang et al., 2022). It is therefore important to illustrate how R can be expanded to account for system‐level resilience and support decision making at a more holistic level. The proposed metric assumes a similar functional form to the system‐level resilience measurements provided in eq. (1) of Ghorbani‐Renani et al. (2020) and eq. (8) of Zhang et al. (2021), which also incorporate a dynamic weighting component that accounts for the time‐dependent importance of infrastructure functionality. However, a crucial difference between these measurements and the metric proposed in this study is the manner in which relative importance is quantified. The Ghorbani‐Renani et al. (2020) and Zhang et al. (2021) approaches measure the importance of functionality in a given infrastructure facility at t relative to that of all other infrastructure facilities within the system or network of interest at the same time (i.e., “inter‐infrastructure” or “facility‐to‐facility” functionality importance; Almoghathawi & Barker, 2019; He & Cha, 2021). These approaches therefore reduce to a time‐independent measurement analogous to $R^{*}$, if only one individual facility is considered. On the other hand, R measures relative functionality importance in an “intra‐infrastructure” sense, that is, the importance of functionality in a given infrastructure facility at t is measured relative to the importance of the same facility at different times. In other words, the Ghorbani‐Renani et al. (2020) and Zhang et al. (2021) approaches are top‐down in nature—where the sets of weightings used across different infrastructure reflect the perspectives or rules of one high‐level (or generic) decision maker in an autocratic process (e.g., “Restoration of the electric power system must be prioritized, because functionality of the water supply system relies on it”)—whereas the R metric is inherently bottom‐up, facilitating bespoke stakeholder priorities related to each unique piece of infrastructure it is applied to (e.g., “Functionality in the electric power system is more important at day 10 than at day 9, because of the increasing risk of fuel depletion in the backup generator with time”).

If necessary, R could be integrated explicitly into a system resilience quantification $R_{sys}$, combining the top‐down and bottom‐up approaches through a formulation such as Cimellaro et al. (2014):

(2) $$R_{sys}=\frac{\sum R_{n}}{N},$$

where N is the number of infrastructure (facilities) within the system of interest. $R_{n}$ is the resilience of the nth facility in the system that could be expressed as an adapted version of R according to:

(3) $$R_{n}=\frac{\int_{t_{0}}^{T_{RE}}Q\left(t\right)w_{n}\left(t\right)w_{sys,n}\left(t\right)dt}{\int_{t_{0}}^{T_{RE}}w_{n}\left(t\right)w_{sys,n}\left(t\right)dt},$$

where $w_{sys,n}$(t) quantifies the nth facility's inter‐infrastructure importance ($0\leq w_{sys,n}\left(t\right)<1$), $w_{n}\left(t\right)$ is equivalent to w(t) (i.e., intra‐infrastructure importance) in Equation (1), and all other variables are as previously defined. To avoid double counting in this case, it is important that $w_{n}\left(t\right)$ is defined independent of the facility's functional interdependencies across the considered system.

## 3 CASE STUDY DEMONSTRATION

**FIGURE 2:**
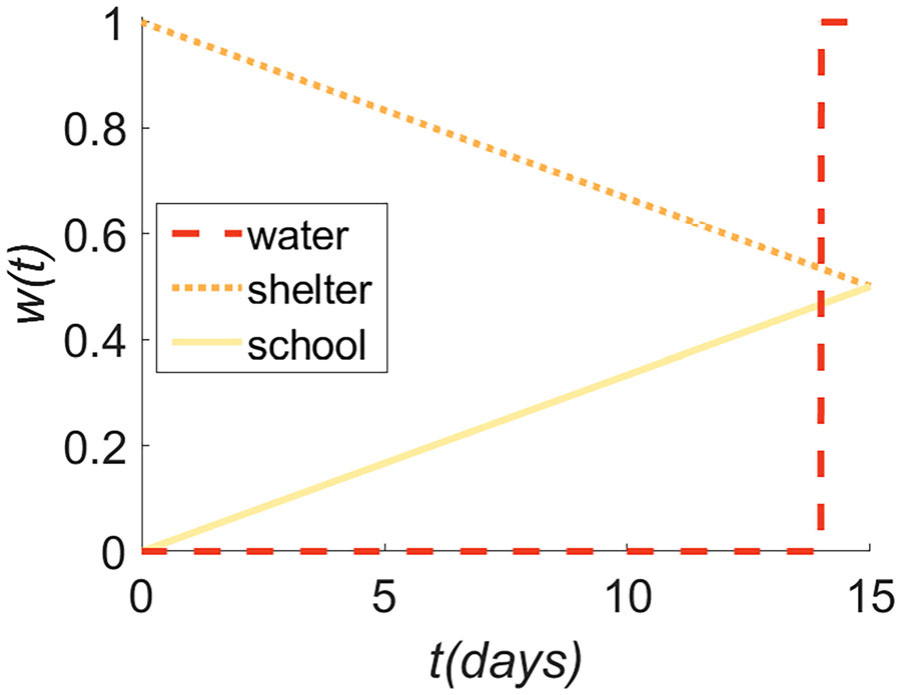
Hypothetical w(t) for a water supply service, an emergency shelter, and a school.

I provide a simple hypothetical case study demonstration of R for three independent infrastructure facilities of interest: a water supply service, an emergency shelter, and a school. I assume that the time of interest is between $t_{0}=0$ and $T_{RE}=15$ days after an “expected” disaster (i.e., a disaster that is reasonably expected to occur once during the life of an urban system, which is typically set as a 50‐year period; Poland, 2009). Hypothetical w(t) values for the three infrastructure, which are plotted in Figure 2 and provided in Table 2, are quantified in line with the PROMETHEE approach, assuming that stakeholders and their associated disaster resilience plans would: (1) consider the importance of functionality in the emergency shelter to decrease over time to zero at t=30 days; (2) deem functionality of the school to be insignificant at $t_{0}$, but increase over time to reach full importance at approximately t=30 days; and (3) assign no importance to functionality of the water supply until t=14 days, which approximately corresponds to the duration of capacity in the backup water system. (1) and (2) are represented using the V‐shape p(x) function with p=30. (3) is described with the U‐shape p(x) function where q=14.

**TABLE 2:** w(t) and resulting R values associated with the hypothetical case study water supply service, emergency shelter, and school, computed for the three hypothetical Q(t). The best R value for each facility is denoted in bold.

Facility	w(t)	$R_{\#1}$	$R_{\#2}$	$R_{\#3}$
Water	$\left\{\begin{array}{cc} 0, & \text{if}\;t\leq 14\\ 1, & \text{otherwise} \end{array}\right.$	0.89	0.82	**0.93**
Shelter	$\left\{\begin{array}{cc} 1-\frac{t}{30}, & \text{if}\;0\leq t\leq 30\\ 0, & \text{otherwise} \end{array}\right.$	0.80	**0.82**	0.81
School	$\left\{\begin{array}{cc} \frac{t}{30}, & \text{if}\;0\leq t\leq 30\\ 1, & \text{otherwise} \end{array}\right.$	**0.88**	0.82	0.86

These functionality requirements approximately reflect some real‐life resilience planning strategies and goals. For instance, after an expected earthquake in the San Francisco Bay area, emergency shelters should be available within 24 h, housing should be restored within 30 to 60 days, and public schools should be open and in session within 30 days (Poland, 2009). Following a magnitude 9 Cascadia Earthquake (with a non‐negligible probability of occurrence during the next 50 years), the Oregon Resilience Plan stipulates that a 30‐day recovery time‐frame for educational institutions is preferable, and that potable water system supplies should be restored within 1 to 2 weeks in the Willamette Valley (Oregon Seismic Safety Policy Advisory Commission, 2013). A 14‐day backup water supply is also in keeping with the emergency water supply planning guidelines of the United States Environmental Protection Agency (American Water Works Association and others, 2011), for instance.

**FIGURE 3:**
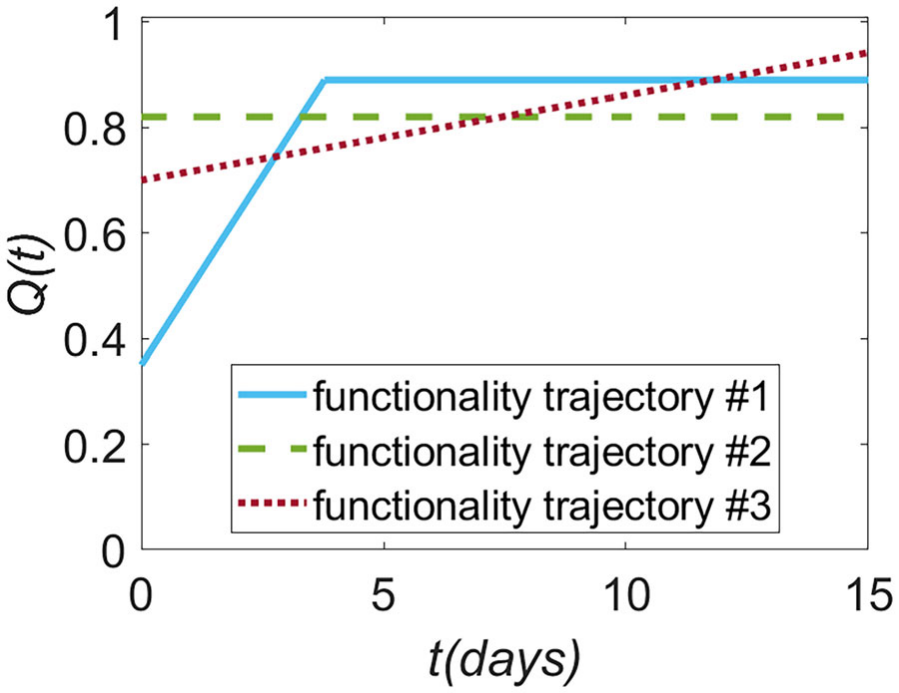
Three hypothetical functionality trajectories Q(t) with identical $R^{*}$ values.

I specifically compare the value of R for three contrasting hypothetical functionality trajectories (see Figure 3) that provide the same value of $R^{*}$, that is,

(4) $$R^{*}=\frac{\int_{0}^{15}Q\left(t\right)dt}{15}=0.82.$$

Trajectory #1 linearly increases from 35% functionality at $t=t_{0}$ to a maximum of 89% functionality at t=3.8 days. Trajectory #3 involves a less steep functionality increase from a higher initial functionality level than trajectory #1 (70%) but reaches 94% functionality within $T_{RE}$. Trajectory #2 remains constant at 82% functionality, independent of time. The R values for each recovery trajectory ($R_{\#1}$ to $R_{\#3}$) and each facility are included in Table 2.

Functionality trajectory #1 produces the highest R value for the school, since it aligns well with the increasing importance of school functionality over time, and provides more functionality than #3 during most days of the post‐disaster period examined. However, the trajectory produces the lowest R value for the emergency shelter, since it provides very little functionality in the immediate aftermath of the disaster.

Functionality trajectory #2 produces identical values of $R=R^{*}$ for each infrastructure facility, since it does not change dynamically. It leads to the highest R value for the emergency shelter and the lowest R value for the water supply service. This is because it provides adequate functionality in the period immediately after the disaster when the shelter is most required, but its functionality is outperformed by that of #1 and #3 when a fully functional water supply service is critical at a later stage.

Functionality trajectory #3 provides the highest value of R for the water supply service. This result is explained by the fact that the trajectory provides the largest functionality (across the three examined trajectories) at the most important time for the water supply service to be operational (i.e., t≥14 days). It is interesting to note that the value of R changes considerably between trajectories for the water supply system; the R value for trajectory #3 is 14% larger than that for trajectory #2 in this case.

In summary, the results indicate that the proposed resilience metric R can distinguish the best functionality trajectory for bespoke infrastructure stakeholder needs, among a set that produces the same level of resilience according to traditional measurements. It is important to note that the results are specific to the considered time period. For example, functionality trajectory #3 provides a higher R value for the school (= 0.93) than trajectory #1 ($R_{\#1}=0.89$) if $T_{RE}=30$ days, given the superior functionality performance of trajectory #3 across the extended time period considered.

## 4 DISCUSSION

By accounting for dynamic end‐user functionality preferences, the proposed metric inherently transforms the concept of resilience quantification from an objective to a subjective measurement. The fundamental implication of this is that two facilities with identical (objective) Q(t) but different (subjective) w(t) are no longer assigned the same resilience value. Resilience is often described as a combination of three capacities: (1) absorptive, (2) adaptive, and (3) restorative (e.g., Vugrin et al., 2010). I argue that the use of w(t) to distinguish between the resilience of facilities with the same Q(t) enables the adaptive capacity element to be more thoroughly captured, as it helps to reveal how well w(t)=1 and Q(t)=1 align; in other words, how well stakeholder functionality planning goals correspond with necessary adjustments in functionality.

An inherent feature of the proposed metric is that it will assign a resilience value of 0 for t values with w(t)=0, regardless of Q(t). Thus, the resilience of a facility that recovers to full functionality relatively quickly could be small if the largest w(t) values occur in the more immediate post‐disaster period. This may seem counterintuitive, but the advantages of this characteristic can be illustrated through a simple example. Assume a facility is required to function as an emergency shelter in the immediate aftermath (i.e., within 2 days) of a disaster and would be replaced with more longer term sheltering arrangements after this time. Setting w(t)=0 (and therefore R=0) for t>2 days captures the specific nuance of the facility being essentially useless (and its resilience meaningless) after only a short period of time.

Furthermore, it is important to note that, similar to the original resilience triangle metric proposed by Bruneau et al. (2003), the proposed metric could produce identical resilience values for very different Q(t). However, this could only happen if: (1) different w(t) values are assigned to the various Q(t) (and therefore the individual Q(t)−w(t) combinations represent separate resilience challenges, for which identical resilience values are likely irrelevant); (2) w(t)=C, meaning that a stakeholder explicitly places equal importance on functionality at all time steps (i.e., they have no dynamic preferences), and identical resilience values for different recovery trajectories would be a reasonable outcome; or (3) there happen to be exactly compensating trade‐offs between Q(t) and w(t) for the different Q(t), which seems unlikely to be a common occurrence. The key strength of the proposed metric over the resilience triangle approach is that it is better able to distinguish the best recovery trajectory for discerning stakeholders with dynamic w(t) (as demonstrated in the case study).

## 5 CONCLUSIONS

This study has proposed a new metric for measuring post‐disaster resilience that explicitly accounts for dynamic fluctuations in the criticality of infrastructure functionality across the post‐disaster period. The time‐dependency of functionality importance is reflected in a dynamic weighting function that can be calibrated through relevant stakeholder feedback, facilitating an end‐user–oriented approach to flexible, context‐specific resilience assessment. The metric is specifically designed for component‐level applications, but could be easily extended to a system‐level context—contributing to the ever‐growing efforts to capture community‐level resilience (Gu et al., 2023)—using some sort of weighted aggregation approach, as discussed in the text.

I have demonstrated the metric using three hypothetical infrastructure components and associated stakeholder input on functionality importance (designed to reflect realistic disaster planning protocols), to identify the best (most resilient) functionality trajectory for each case, among a synthetic set of three. Each of the investigated functionality trajectories yield the same resilience value computed according to the traditional triangular‐based metric first introduced by Bruneau et al. (2003), despite having significantly different shapes. On the contrary, the proposed metric provides reasonably different values for the trajectories, in line with stakeholder functionality requirements. For instance, the highest resilience value is assigned to the trajectory with the most initial post‐disaster capacity if stakeholders prioritize emergency‐phase functionality (i.e., in the case of an emergency shelter), whereas trajectories with maximum functionality later on in the recovery process produce the highest resilience values if stakeholders do not perceive immediate functionality to be essential (i.e., in the case of a school or a water supply for which there are temporary backup resources).

Although the case study demonstration is hypothetical in nature and does not involve real stakeholder feedback, it still indicates that the metric can naturally distinguish diverse optimum recovery trajectories for different infrastructure, based on bottom‐up underlying stakeholder needs rather than (at least exclusively) relying on top‐down autocratic comparisons of functionality importance across different types of infrastructure. This is a useful feature of the proposed metric that could be leveraged to effectively coordinate the post‐disaster recovery process across different types of infrastructure and various relevant stakeholders (e.g., civic agencies, utility infrastructure operators, and nongovernmental organizations) in a given urban system, in the face of limited recovery resources, investment, and time (e.g., Olshansky et al., 2012; Choi et al., 2019; Pant et al., 2014). This type of coordination process would first involve designing a series of bespoke recovery trajectories that account for unavoidable constraints (e.g., construction worker shortages) across time. The proposed metric could then be used to appropriately assign each trajectory to a corresponding infrastructure facility, in accordance with the dynamic importance of its functionality. The metric is similarly useful for facilities with multiple purposes over time; in this case, it can help to determine suitable functionality trajectories for each use. In summary, the proposed metric for post‐disaster resilience quantification across individual facilities possesses promising potential as an effective tool for facilitating informed stakeholder‐oriented decision making on post‐disaster infrastructure recovery. Future work will focus on applying the metric to more expansive case studies involving real stakeholders and exploring its extension to a more explicit consideration of system‐level resilience.

## CONFLICT OF INTEREST STATEMENT

The author has no competing interests to declare.
